# Antagonistic Effect of Plant Growth-Promoting Fungi Against *Fusarium* Wilt Disease in Tomato: In vitro and In vivo Study

**DOI:** 10.1007/s12010-022-03975-9

**Published:** 2022-06-11

**Authors:** Mohamed S. Attia, Deiaa A. El-Wakil, Amr H. Hashem, Amer M. Abdelaziz

**Affiliations:** 1 Botany and Microbiology Department, Faculty of Science, Al-Azhar University, Cairo-11884, Egypt; 2 Department of Biology, Faculty of Science, Jazan University, Jazan, 82817 Saudi Arabia; 3 Plant Pathology Research Institute, Agricultural Research Center, Giza, 12619 Egypt

**Keywords:** Plant growth-promoting fungi, *Fusarium oxysporum*, Antifungal activity, Phytopathology, Induction of systemic resistance, Biological control

## Abstract

Fusarium wilt is considered one of the most destructive diseases for tomato plants. The novelty of this work was to investigate the antifungal and plant growth-promoting capabilities of some plant growth-promoting fungi (PGPF). Plant growth-promoting fungi (PGPF) improved the plant health and control plant infections. In this study, two fungal strains as PGPF were isolated and identified as *Aspergillus fumigatus* and *Rhizopus oryzae* using molecular method. The extracts of *A. fumigatus* and *R. oryzae* exhibited promising antifungal activity against *F. oxysporum* in vitro*.* Moreover, antagonistic effect of *A. fumigatus* and *R. oryzae* against *F. oxysporum* causing tomato wilt disease was evaluated in vivo. Disease severity and growth markers were recorded and in vitro antagonistic activity assay of the isolated *A. fumigatus* and *R. oryzae* against *Fusarium oxysporum* was measured. Physiological markers of defense in plant as response to stimulate systemic resistance (SR) were recorded. Our results indicated that *A. fumigatus* and *R. oryzae* decreased the percentage of disease severity by 12.5 and 37.5%, respectively. In addition, they exhibited relatively high protection percentage of 86.35 and 59.06% respectively. *Fusarium* wilt was declined the growth parameters, photosynthetic pigments, total soluble carbohydrate, and total soluble protein, whereas content of free proline, total phenols, and the activity of antioxidant enzymes activity increased under infection. Moreover, application of *A. fumigatus* and *R. oryzae* on infected plants successfully recovered the loss of morphological traits, photosynthetic pigment total carbohydrates, and total soluble proteins in comparison to infected control plants. PGPF strains in both non-infected and infected plants showed several responses in number and density of peroxidase (POD) and polyphenol oxidase (PPO) isozymes.

## Introduction

Tomato diseases acutely affect its crop and accordingly considered of great economic importance [1]. There are many destructive diseases of both quality and quantity of tomato production [2]. Under the threat of climate changes and the widespread of pathogens, improving crop productivity and avoiding the use of chemical pesticides is a major issue for the agricultural industry [3]. However, *Fusarium* wilt disease mainly caused by *Fusarium oxysporum* is affecting severe injury through all phases of plant growth [4]. Lately, in Egypt, the injuries in tomato production due to *F. oxysporum* infection raised up to 67% of total planted area that makes severe damage during all stages of plant development [5]. The traditional strategies to limit the disease, the use of antifungal compounds, and crop cycle have not been effective due to spores can stay viable for numerous years and the harmful effects of pesticide residues on human health. Thus, it is necessary to improve new and efficient control strategies that do not affect the environmental safety [6].

Biological control is an alternative to chemical control of the *Fusarium* wilt diseases through antagonist nonpathogenic organisms that have potency to reduce the harmful effects of *Fusarium* wilt in several crops [7]. Recent studies powerfully favored application of biological agents as safety approaches for human and environment to control *F. oxysporum* in Egypt [8]. Stimulated resistance is a physiological state of protection potency produced by a specific eco-friendly stimuli that acts essential role against a broad range of plant pathogens including fungi [9]. Plant growing can be simply stimulated by fungi through several mechanisms, such as systemic resistance’s stimulation, plant nutrition enhancements, and via their toxicity to various pathogens [10, 11]. Many plants’ rhizosphere was used for the isolation of several microbial strains have antagonistic activity. Plant growth-promoting fungi can produce chemical compounds with different benefits for the plant. Among them, HCN which was recognized as a bio-control agent, based on its ascribed toxicity against plant pathogens [12, 13]. HCN is a broad-spectrum antifungal compound playing a vital role in the bio-control of fungal disease as has been demonstrated in several studies [12–15]. Moreover, Ramette and Frapolli [16] proposed that HCN compound works on the cells of the pathogen by obstructive the cytochrome oxidase of the respiratory chain. PGPF produced were able to produce IAA. Also, IAA works a vital role in the improvement of plants by stimulating their growth when applied directly to the roots [82]. PGPF were able to solubilize organic phosphates which play a role in enhancement plant health [17, 18]. Herein, this study aimed to investigate the capabilities of PGPF on the growing of tomato diseased with *F. oxysporum* in vitro as well as in vivo. Our study opens the approach to an alternative and safety techniques to control the *Fusarium* wilt disease in tomato. We believe this study poses a great value and importance to integrate *Fusarium* wilt management.

## Materials and Methods

### Tomato Plant

Well-identified 4-week-old tomato seedlings (*Solanum Lycopersicon* L. cv. Castlerock II PVP) were obtained from Agricultural Research Center (ARC), Ministry of Agriculture, Giza, Egypt.

### Source and Maintenance of the Fungal Pathogen

*F. oxysporum f. sp. Lycopersici* RCMB008001 was obtained from Regional Center for Mycology et al.-Azhar University. Then it was confirmed by the pathogenicity test according to Hibar, Edel‐Herman [19]. The inoculum of the pathogen was prepared according to Aldinary and Abdelaziz [20].

### Source, Isolation, and Identification of PGPF from Rhizosphere

Rhizosphere was collected from plant field (10 g). Then, 90-ml sterile distilled water was used to make a suspension. Serial dilution technique was performed from 10^–2^ to 10^–6^. Aliquots of 0.1 ml were spread on sterile Petri dishes containing sterilized Potato Dextrose Agar (PDA) medium amended with chloramphenicol (200 µg/L) [21, 22]. The Petri dishes were incubated for 3–7 days at 30 °C [19, 23–25].

Fungal isolates were identified depending on their morphological characteristics according to recent studies [26–31]. Macroscopic morphological features including color, texture, diameter of colonies, and microscopic characteristics including vegetative and reproductive structures of the fungi were noted. Then fungal isolates were identified genetically using ITS gene. The genomic DNA was isolated and purified using Quick-DNA Fungal Microprep Kit (Zymo research; D6007), and molecular identification was achieved by internal transcribed spacer (ITS) region [32–37].

### In vitro Antagonistic Activity of PGPF Against F. oxysporum

Well diffusion method was applied to study the antifungal activity of ethyl acetate fungal extracts of *A. fumigatus* and *R. oryzae*. *F. oxysporum* was inoculated on PD broth medium, then incubated at 28 ± 2 °C for 3–5 days. Fungal inoculum of *F. oxysporum* was spread thoroughly on the sterilized solidified potato dextrose agar (PDA) medium. Wells (7 mm) were filled with 50 µl of each fungal extract (4 mg/ml) were put in each well. The culture plates were incubated at 25 °C for 7 days, and the zones of inhibition were observed and measured. Moreover, minimum inhibitory concentration (MIC) was carried, where different concentrations of each fungal extract (4, 2, 1, 0.5 and 0.25 mg/ml) were put in wells to detect MIC.

### Pot Experiment: In vivo Study

Applied elicitors (PGPF) were added 1 week before infection with *Fusarium oxysporum*. The pot trials were conducted at the experimental farm of Botany and Microbiology Department, Faculty of Science, Al-Azhar University. Seedlings were planted in six groups as following:

(1) plants without any treatment were referred to as healthy control, (2) plants infected with *Fusarium oxysporum* as infected control, (3) healthy plants treated with *A. fumigatus* strain, (4) infected plants treated with *A. fumigatus*, (5) healthy plants treated with *Rhizopus oryzae*, (6) and infected plants treated with *Rhizopus oryzae*. Disease development and severity were recorded 15 days post inoculation. The plant samples were collected for morphological and biochemical indicators for resistance analysis when the plants were 60 days old.

### Disease Symptoms and Disease Index

Disease symptoms and PGPF protection percent were assessed according to Farrag, Attia [8]. The disease symptoms were observed, and disease severity and the protection percentage of PGPF were estimated by the equation: disease index (DI) was calculated using the five-grade scale according to the formula: DI = (1n_1_ + 2n_2_ + 3n_3_ + 4n_4_)100/4n_t_ and protection % = A–B/A × 100%, where, n1-n4 are the number of plants in scales and net total number of tested plants, A = PDI in infected control plants B = PDI in infected-treated plants.

### Metabolic and Biochemical Indicators for Plant Resistance

Quantitative determination of pigments was carried out according to the method used by Vernon and Seely [38], while the well-established method of Lowry, Rosebrough [39], using casein as a standard protein was used to determine the total soluble proteins. In addition, the soluble carbohydrate content of the dried shoot was calculated by the method used by Irigoyen, Einerich [40], while the phenolic compounds were determined according to method used by Diaz and Martin [41]. In addition, free proline content was evaluated [42]. Peroxidase activity was determined according to Srivastava [43]. Also, the adopted method from Matta and Dimond [44] was followed for measuring the activity of polyphenol oxidase.

### Statistical Analyses

Analysis of experimental data was achieved by using one-way analysis of variance (ANOVA), while means differences were separated using Duncanʼs multiple range test and the (LSD) at 5.0% level of probability following Costate Software [45, 46].

## Results

### Isolation and Identification of Fungal Isolates

**Fig. 1:**
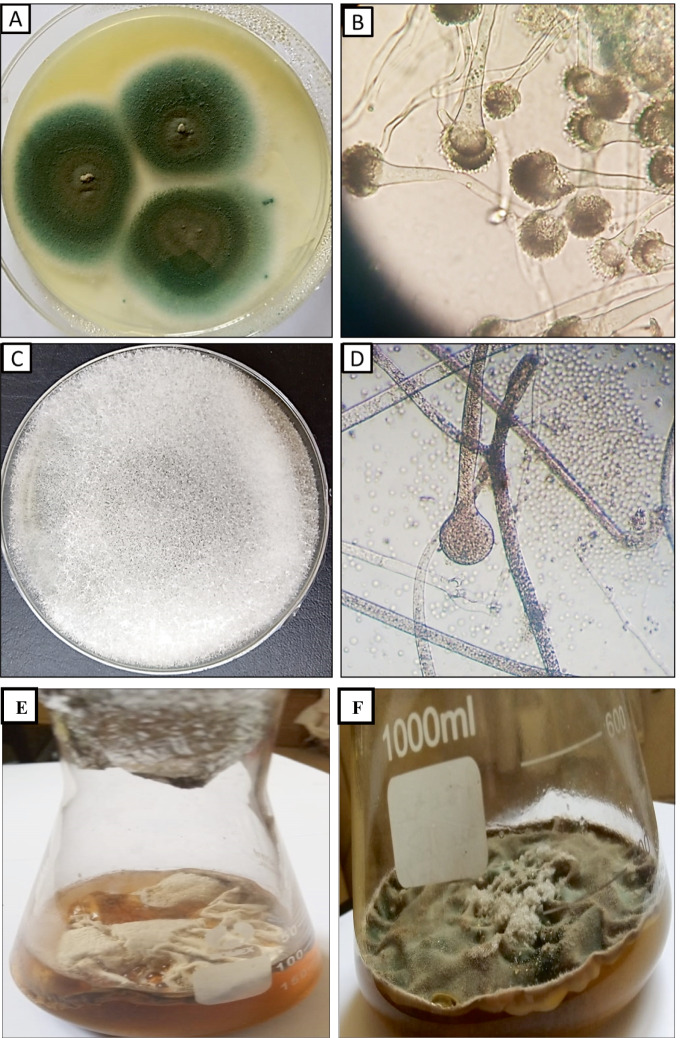
**A** Colony of *A. fumigatus* on PDA grown at 28 °C for 4 days showing the culture characteristics. **B** Light microscope showing rough walled conidia, stipe, conidia, sterigmata, and conidial head of *A. fumigatus* (400X). **C** Colony of *R. oryzae* on PDA grown at 28 °C for 4 days showing the culture characteristics. **D** Light microscope showing sporangiophores, sporangium, sporangiospores, and rhizoids of *R. oryzae* (100X):1. **E** Growth of *R. oryzae* on PD broth medium and **F** growth of *A. fumigatus* on PD broth medium grown at 28 °C for 15 day

**Fig. 2:**
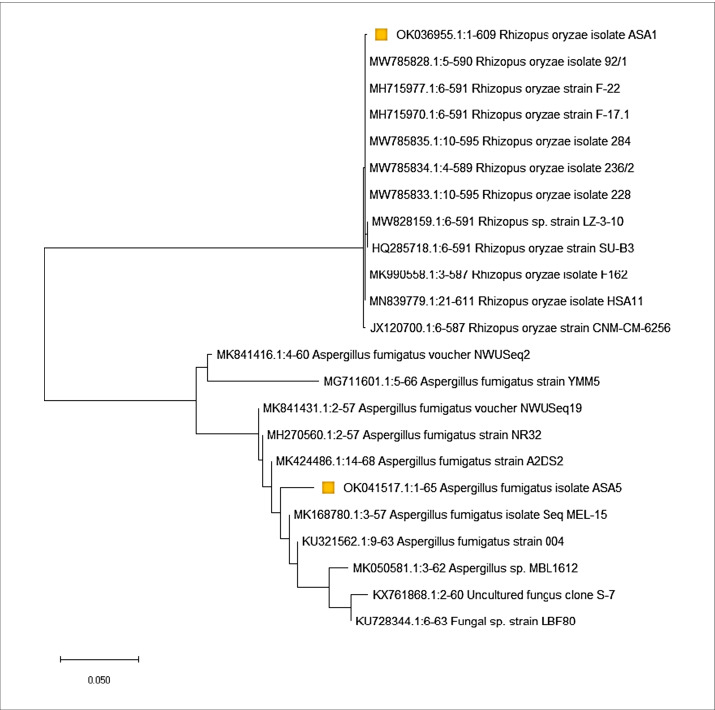
Phylogenetic tree of *A. fumigatus* and *R. oryzae* in relative with international isolates

Two fungal isolates A1 and A5 were isolated from soil sample collected from Tamiya, Fayoum Governorate, Egypt. The two fungal isolates were identified morphologically and genetically. Morphologically, the fungal isolate A1 was identified as *Rhizopus oryzae* where colonies are fast growing andaare white in color; sporangiophores are brownish and branched; and sporangia are black in color and spherical in shape (Fig. 1A & B). On the other side, fungal isolate A5 was identified as *Aspergillus fumigatus* where colonies grow rapidly reaching 3.0–5.0 cm diameter in 4 days at 28 °C on PDA medium, showing rapid rate of growth with smoky grayish green in color, oval vesicle, bearing single series of sterigmata covered mostly half of the vesicle, conidial head is a columnar, dome shape (Fig. 2C & D). Molecular identification using ITS gene confirmed that A1 and A5 is resemble to *Rhizopus oryzae* and *A. fumigatus* with similarity 99%, respectively. The sequences of the two strains *Rhizopus oryzae* and *A. fumigatus* were recorded in GenBank with accession numbers OK036955 and OK041517, respectively. (Fig. 2).

### In vitro Antifungal Activity of PGPF Strains Against F. oxysporum

**Fig. 3:**
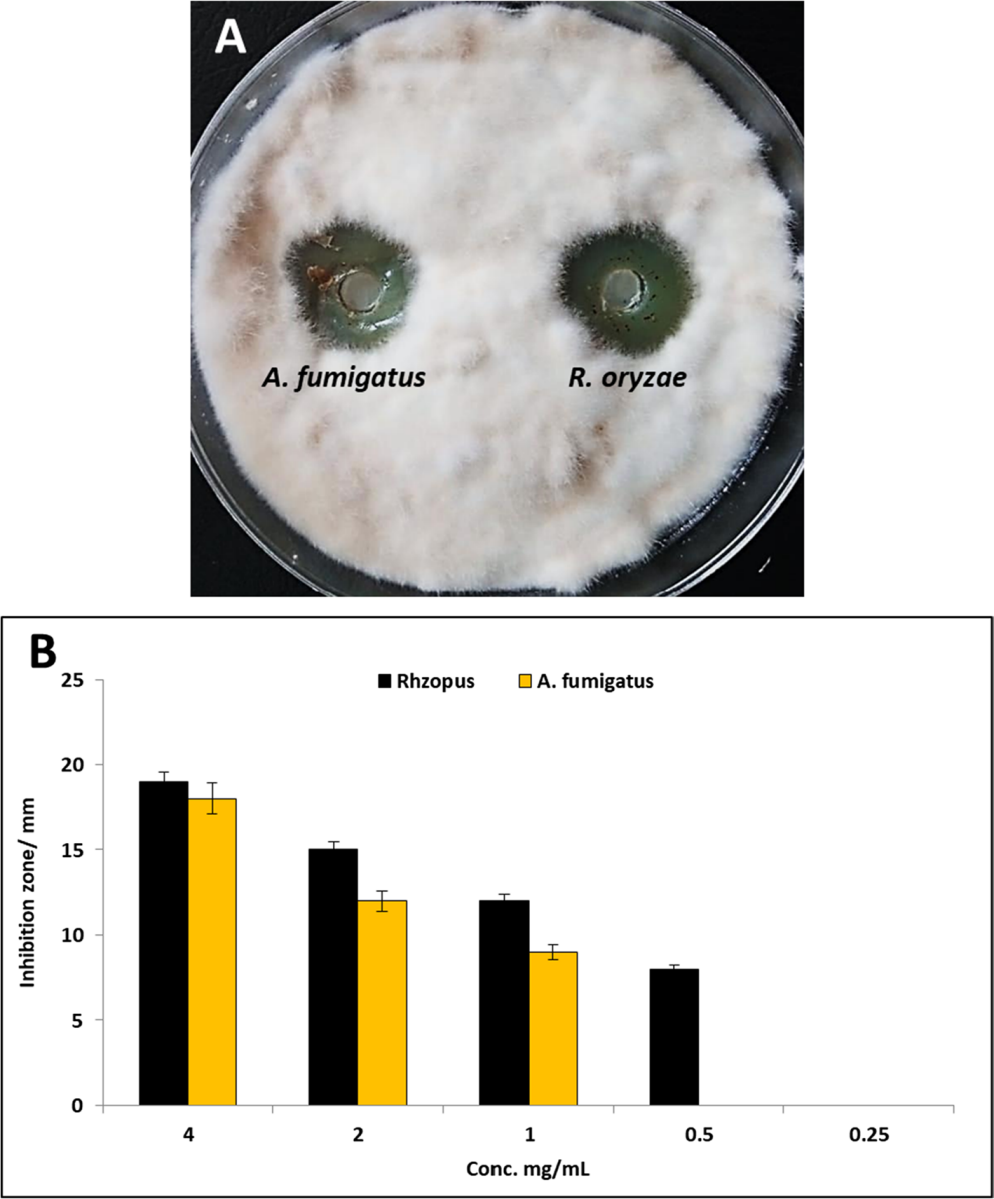
Antifungal activity of *A. fumigatus* and *R. oryzae*: A) Inhibition zone; B) MIC

Antifungal activity of fungal extracts of *R. oryzae* and *A. fumigatus* was evaluated against *F. oxysporum* using agar well diffusion method as shown in Figure 3. Results revealed that the two fungal extracts exhibited a promising antifungal activity against *F. oxysporum*, where inhibition zones of *A. fumigatus* and *R. oryzae* at concentration 4 mg/ml were 18 and 19 mm, respectively (Figure 3A). Moreover, different concentrations (4.0 – 0.25 mg/ml) of the two extracts were evaluated to detect MIC for each extract as shown in Figure 3B. Results illustrated that MIC of *R. oryzae* extract was 0.5 mg/ml, while MIC of* A. fumigatus* extract was 1 mg/ml.

### Parameters Evaluation and Estimation of Systemic Resistance Induced by PGPR Against F. oxysporum Caused Tomato Wilt Disease

#### Disease Severity (DS) and Protection Percent

Results in Table 1 indicated that *A. fumigatus* and *R. oryzae* were reducing the percentage of disease severity by 12.5 and 37.5%, respectively. In addition, they exhibited high protection percentage of 86.35 and 59.06%, respectively with regard to control.

**Table 1:** Effect of PGPF on disease index of infected Tomato plants

Treatment	Disease symptom classes					DI (disease index) (%)	Protection (%)
	0	1	2	3	4		
Control healthy	6	0	0	0	0	0	-
Control infected	0	0	0	2	4	91.6	0
Infected + *A. fumigatus*	3	3	0	0	0	12.5	86.35
Infected + *R. oryzae*	2	1	1	2	0	37.5	59.06

#### Growth Indicators

**Fig. 4:**
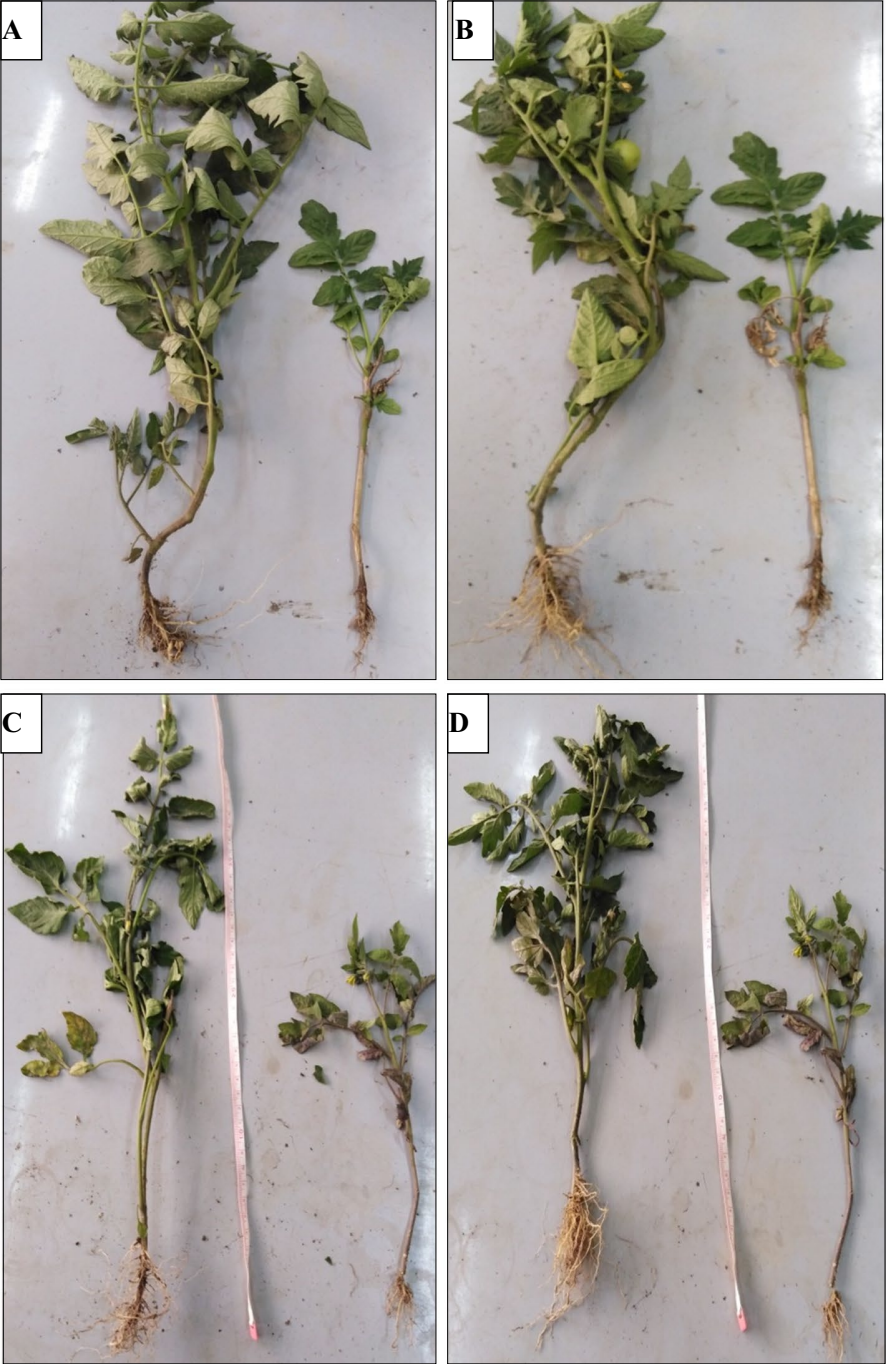
Effect of PGPF on morphological indicators of tomato plants. **A** Healthy + *R. oryzae.* **B** Healthy + *A. fumigatus.* **C** Infected + *A. fumigatus.* **D** Infected + *R. oryzae*

It is clear from data in Table 2 and Fig. 4, various growth parameters (shoot length, root length, and number of leaves) were significantly improved by application of *A. fumigatus and R. oryzae*. Tomato plants infected with *F. oxysporum* showed significant loss of shoot length, root length, and the number of leaves. Moreover, the loss of shoot length (48.23%), root length (51.16%), and number of leaves (78.07%) in comparison to healthy control plants. On the other hand, the application of *A. fumigatus* and *R. oryzae* on infected plants successfully recovered the loss of shoot length, root length, and number of leaves in comparison to infected control plants.

**Table 2:** Morphological indicators of tomato plant treated with PGPF in vivo conditions

Treatment	Shoot length (cm)	Root length (cm)	No. of leaves/plant
Control healthy	47 ± 2^b^	27.5 ± ^b^	152 ± ^c^
Control infected	24.33 ± 1.5^d^	13.43 ± 0.45^c^	33.33 ± 1.15^f^
Healthy + *A. fumigatus*	57.33 ± 1.25^a^	33.1 ± 4.5^a^	186.66 ± 2.08^a^
Infected + *A. fumigatus*	46.5 ± 0.7 ^b^	26 ± 0.49^b^	124 ± 1^d^
Healthy + *R. oryzae*	56 ± 1^a^	30 ± 1^ab^	159.5 ± 2.12^b^
Infected + *R. oryzae*	45 ± 2.01^b^	16.7 ± 0.43^c^	82.33 ± 2^e^
LSD at 0.05	4.876	6.28	5.01

Significance power a>b>c>d>e

#### Photosynthetic Pigments

As shown in Table 3, chlorophyll a and b contents were significantly decreased in the infected plants. On the other hand, infected plant treated with tested elicitors (*A. fumigatus* and *R. oryzae*) showed a significant enhance in the content of chlorophyll a, b compared to the infected control. Also, the treatment with *A. fumigatus* exhibited the most potent effect in terms of the chlorophyll a and b contents than plants treated with *R. oryzae*, compared to the non-treated infested control. However, when healthy plants treated with tested elicitors *A. fumigatus* and *R. Oryzae,* a promising recovery response in comparison to healthy control plants was observed. Additionally, the contents of carotenoids were significantly increased in tomato plant in response to *F. oxysporum* infection. In *Fusarium-*infected plant and treated with *A. fumigatus* and *R. Oryzae,* the contents of carotenoids were markedly increased compared to the non-treated infested control.

**Table 3:** Photosynthetic pigments of tomato plant treated with PGPF in vivo conditions

Treatment	Chlorophyll a (mg/g fresh weight)	Chlorophyll b (mg/g fresh weight)	Carotene (mg/g fresh weight)
Control healthy	21.06 ± 0.75^bc^	13.04 ± 0.22^ab^	1.77 ± 0.1^c^
Control infected	15.44 ± 1.32^d^	6.6 ± 0.42^d^	2.99 ± 0.14^a^
Healthy + *A. fumigatus*	23.93 ± 1.18^a^	13.43 ± 0.63^a^	3 ± 0.11^a^
Infected + *A. fumigatus*	20.12 ± 0.73^bc^	12.06 ± 0.5^b^	3.22 ± 0.03^a^
Healthy + *R. oryzae*	22.55 ± 0.26^ab^	10.1 ± 0.38^c^	2.87 ± 0.105^a^
Infected + *R. oryzae*	19.8 ± 1.16^bc^	9.04 ± 0.77^c^	2.2 ± 0.18^b^
LSD at 0.05	3.09	1.53	0.39

#### Physiological and Metabolic Changes

**Fig. 5:**
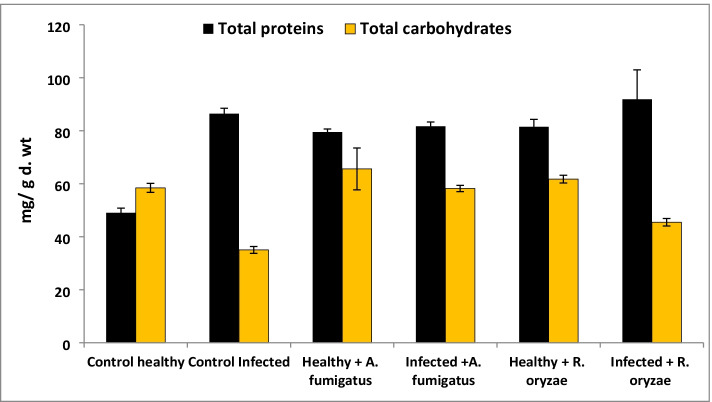
Effect of PGPF on total carbohydrate and protein of tomato plants

**Fig. 6:**
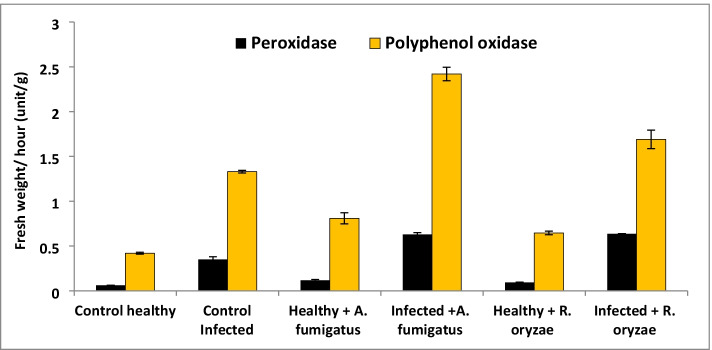
Effect of PGPF on enzyme activity of tomato plants

Results reveled that total soluble proteins and total carbohydrate of tomato decreased significantly in response to the infection with *F. oxysporum* (Fig. 5). On the other hand, *A. fumigatus* or *R. oryzae* led to significant increase in the contents of total soluble proteins of infected plants. However, pre-treatment with *R. oryzae* resulted in significant effect in terms of the total soluble protein and total carbohydrate contents more than *A. fumigatus* and the non-treated-infected plants. The total phenols and free proline of tomato increased significantly in response to *F. oxysporum* infection (Table 4). It is noticeable that the greatest value recorded for the total phenols and free Proline was achieved by applied *R. oryzae* followed by *A. fumigatus*. Moreover, Results in Fig. 6 revealed that the changes in the activities of oxidative enzymes (Peroxidase; POD and Polyphenol Oxidase; PPO) in infected plants were significantly increased with respect to the non-infected plants (control). Additionally, the most significant increase in POD activity was achieved by utilizing *Rhizopus oryzae* on the infected plants followed by *A. fumigatus*. While, the most significant increase in PPO activity was achieved by using *A. fumigatus* on the infected plants followed by *Rhizopus oryzae* compared to infected tomato plants.

**Table 4:** Metabolic indicators of tomato plant treated with PGPF

Treatment	Total phenol (mg/g d. wt)	Total prolin (mg/g d. wt)
Control healthy	0.55 ± 0.13^d^	0.12 ± 0.003^e^
Control infected	3.39 ± 0.1^a^	0.52 ± 0.02^b^
Healthy + *A. fumigatus*	1.48 ± 0.04^c^	0.18 ± 0.006^d^
Infected + *A. fumigatus*	2.8 ± 0.34^ab^	0.50 ± 0.008^b^
Healthy + *R. oryzae*	1.94 ± 0.14^bc^	0.23 ± 0.018^c^
Infected + *R. oryzae*	3.47 ± 0.83^a^	0.95 ± 0.03^a^
LSD at 0.05	1.136	0.062

Significance power a>b>c>d>e

#### Isozymes

##### POD Isozymes

**Fig. 7:**
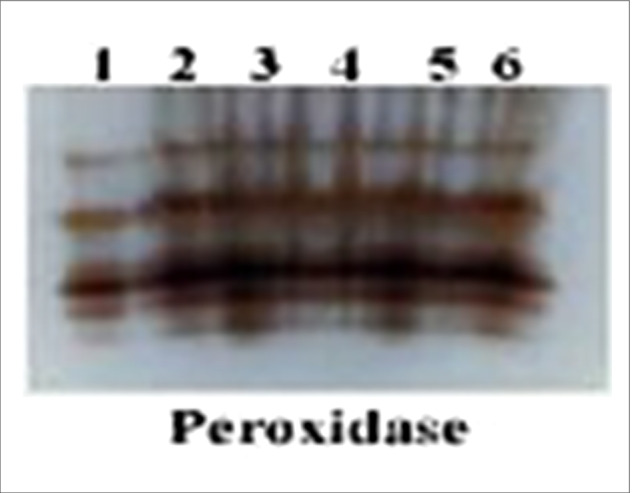
Effect of *F. oxysporum* and application of *A. fumigatus* and *R. oryzae* on peroxidase isozyme of tomato plants

Native PAGE in Fig. 7 and Table 5 showed seven POD isozymes at Rf (0.484, 0.607, 0.806, 0.855 and 0.934). *Fusarium* -infected plants showed highly over expressed POD that recorded 5 bands including 3 faint bands at Rf (0.484, 0.607and 0.934), 1 moderate bands at Rf (0.806) and 1 highly dense band at Rf (0.855). Application *A. fumigatus* or *R. oryzae* on infected plants recorded the same 5 bands at the same Rf in which 3 of them were faint bands at Rf (0.484, 0.607and 0.934), while the other 2 bands were moderate t at Rf (0.806, 0.855). Healthy plants treated with *A. fumigatus* or *R. oryzae* expressed the lowest POD expression that they produced 2 faint bands at Rf (0.484 and 0.607) and 1 moderate band at (0.806).

**Table 5:** Isomers of peroxidase enzymes (+ / −) and their Retention factor (Rf)

RF	Lane 1	Lane 2	Lane 3	Lane 4	Lane 5	Lane6
0.484	+	+	+	+	+	+
0.607	+	+	+	+	+	+
0.806	+ +	+ +	+ +	+ +	+ +	+ +
0.855	+	+ + +	-	+ +	-	+ +
0.934	+	+	-	+	-	+

L1 = Control, L2 = Control Infected, L3 = Healthy + *A. fumigatus*,

L4 = Infected + *A. fumigatus*, L5 = Healthy + R. oryzae, L6 = Infected + R. oryzae.

##### PPO Isozymes

**Fig. 8:**
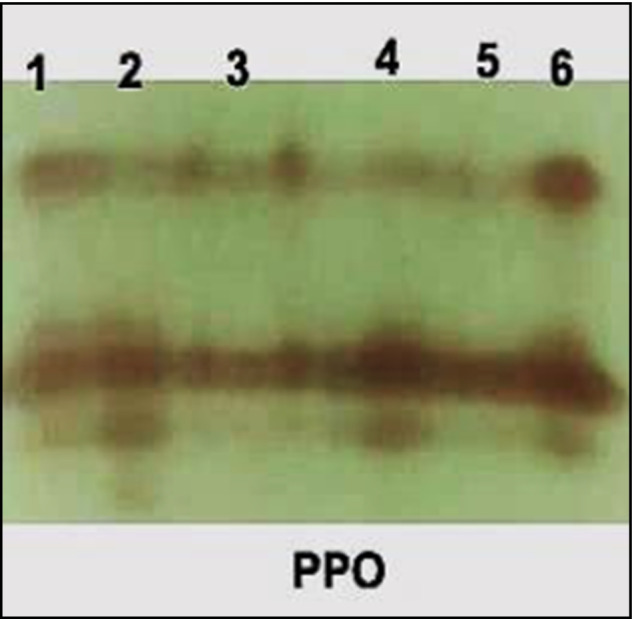
Effect of *F. oxysporum* and application of tested elicitors (*A. fumigatus* or *R. oryzae*) on peroxidase isozyme of tomato plants

The PPO isozyme of plant leaves showed three PPO isozymes at Rf (0.204, 0.629 and 0.786) in Fig. 8 and Table 6. Fusarium -infected plants showed the highly PPO expression that produced 3 bands including 2 moderate bands at Rf (0.204 and 0.786), 1 highly dense band at Rf (0.629). Under fusarium infection conditions, treatment with *A. fumigatus* recorded 3 faints bands at Rf (0.204, 0.629 and 0.786) and 1 moderate band at Rf (0.786), while *Rhizopus oryzae* treatment gave a high expression of PPO resulted in 1 moderate bands at Rf (0.204), 1 faint bands at Rf (0.786), and 1 highly dense band at Rf (0.629).

**Table 6:** Isomers of polyphenol oxidase enzymes (+ / −) and their retention factor (Rf) in response to *Fusarium*

RF	Lane 1	Lane 2	Lane 3	Lane 4	Lane 5	Lane 6
0.204	+ +	+ +	+	+	+	+ +
0.629	+ +	+ + +	+ +	+ + +	+ +	+ + +
0.786	+	+ +	-	+ +	-	+

## Discussion 

It is well recognized that defiance to pathogens can be improved within plants through exogenous use of biotic or abiotic agents. Non-pathogenic rhizo-fungi and their metabolites are considered one of the most important biotic elicitors [47, 48]. The application of PGPF resulted to induct plant growth as well as induced systemic resistance responses to biotic stresses [49]. Application of natural agents for controlling of fungal phytopathogens as safe agents for soil micro flora living organisms and the environment instead of chemical fungicides. For this reason, in this study, two fungal strains *A. fumigatus* and *R. oryzae* as PGPF which could be used to generate plant defiance against *Fusarium* wilt. In accordance with our results, previous studies reported that *Aspergillus* species can be used for growth promotion and control of fungal plant diseases [50–54]. Hung and Lee Rutgers [50] illustrated that *Aspergillus* spp. induce the plant growth through the production of active compounds. Another study, *Aspergillus* isolated from rhizosphere of wheat produced multiple plant growth inducers as IAA, GA, and siderophores, that resulted in phosphate solubilization, enhancement seed germination percent, and plant height [51]. Furthermore, *R. arrhizus* KB-2 was used as plant growth promoter through production of gibberellin, indole acetic acid, and abscisic acid [55]. The first indicator to systemic resistance incidence in plants was the treatment with PGPF which minimized the DS % as well as established the protection against *F. oxysporum*. According to the presented data, the treatment with *A. fumigatus* strain was the best treatment in terms of reducing the PDS and recorded the highest protection. These explained by Jovičić-Petrović and Jeremić [56] which reported that *Aspergillus* established inhibition percentage against *F. oxysporum* by (33%). Also Kriaa and Hammami [57] recorded *Aspergillus* has antifungal activity against *Fusarium*; thus it applied as a new bio fungicide. Also, Peeran and Prasad [58] proved the efficient antifungal activity of *R. oryzae* against plant pathogens. Furthermore, Kang and Hong [59] studied the antifungal activity of *Aspergillus* against *Phytophthora* phytopathogens. Also, [20] reported that *Aspergillus* is applied as effective biological control agents againstt*F. oxysporum*. For more Espinoza, González [60] reported that *Rhizopus* has fungicidal activity like that of fungicide Captan against wide range of plant pathogens. *Aspergilli* species are able to produce a great number of bioactive secondary metabolites as bioactive proteins, enzymes that may be resulted in plant recovery from harmful effect of *Fusarium* infection [61]. *Rhizopus oryzae* the has ability to produce the supportable platform chemicals lactic acid, fumaric acid, and ethanol that may be promoting the plant growth and enhancement the soil properties [62].

Many microbial functions of *Aspergillus* has been reported, through stimulation of ionic transport to enhancement vegetative growth and production of aminocephalosporanic acid acylase enzymes [63]. In this study, the tomato growth parameters (shoot length, root length, number of leaves per plants, chlorophyll and carbohydrates) were significantly decreased due to *Fusarium* infection. In this respect, this decreasing may be associated with the disorders in the distribution of the growth regulating hormones [4, 64–68]. Our results proved that, the treatment of infected tomato plant with *A. fumigatus* and *R. oryzae* led to significantly improve the plant growth characters compared to control plants. Our results are in harmony with those informed by Alwathnani, Perveen [69]. Moreover, these improved all growth parameters compared with control. These results explained by production of secondary metabolites that induce the growth of plants under stress conditions [70].

Photosynthesis is the main purpose of plants, empowering them to convert light energy into chemical energy which next utilized in all cell activities and it is highly altered by pathogenic infection [71, 72]. In the current study, *F. oxysporum* caused a significant decreasing in both photosynthesis, resulting in inhibition of growth. The decline in chlorophyll was well described by Kyseláková and Prokopová [73] which reported that infection may be resulted to the oxidative stress that caused damage chlorophyll a; this means that the plant fail in bagging sunlight and thus photosynthesis will be reduced or inhibited. Pigment contents were positively affected due to treatment with *A. fumigatus* and *R. Oryzae*; this result became one of the visible pieces of evidence of treatment efficiency. Our study revealed that treatments with *A. fumigatus* and *R. oryzae* showed significant increase in the pigment contents compared with control. Our results are harmony with those reported in the literature [65, 66, 74]. The positive effect in photosynthetic pigments due to treatment of *A. fumigatus* and *R. oryzae* may be attributed to enriching the plant and its soil with N_2_ element. Our results agreed with Farrag and Attia [8]*;* they reported that the totalssoluble protein increased significantly due to *Fusarium* infection. These results explained by Nafie [75] who recorded that *F. oxysporum* infection induced the plant to form of nitrogenous constituents. The indirect effects of PGPF strains in the disease suppression include the activation of the plant defense mechanisms through the production of proteins when tested with pathogens [76]. For more soluble sugars involved in the responses to a number of stresses may be resulted in modifications of gene expression [77]. Total phenols act as scavengers agents for free radical and substrate for many antioxidant enzymes [78]. Our results showed that total phenol of infected tomato seedlings was significantly increased. However, the application of *A. fumigatus* and *R. oryzae* resulted in different responses in both total phenols and free proline of plants. On the other hand, high values of total phenols and free proline were achieved. Total phenols play a vital key in plant metabolic regulation, plant growth, and the lignin production [79]. Our results showed that proline contents significantly increased in plants treated with *A. fumigatus* and *R. oryzae*. These results are in agreement with Gupta [80] and Al-Wakeel, Moubasher [81]; they reported that the proline contents significantly increased during the fungal pathogenesis.

The highest increase in POD and PPO activities were determined due to the treatment with *A. fumigatus* and *R. oryzae*. This enhancement of PPO activities against disease have been recorded [82]. Protein profile showed seven POD isozymes and four PPO isozymes sign the extract of leaf-soluble proteins. Many new isozyme bands were induced by *Fusarium* infection thus the antioxidant enzyme activities in *Fusarium* -infected plants treated with *A. fumigatus* and *R. oryzae* were better than those in control plants. These results explained the major role of our fungal strains (*A. fumigatus* and *R. oryzae*) as a plant growth promotors and isolates in protecting tomato plants against *Fusarium* wilt disease.

## Conclusion

The present investigation conducted a new method focused on application of plant growth promoting fungi in the induction of the systemic resistance against fungal plants diseases. The isolated PGPF were conducted to the molecular characterization and identified as *A. fumigatus* and *R. oryzae*. In vitro, both *A. fumigatus* and *R. oryzae* exhibited potential antifungal activity *F. oxysporum* causing *Fusarium* wilt disease. In vivo, application of *A. fumigatus* and *R. oryzae* for 1 week before *Fusarium* infection showed positive effect in plant growth parameters including plant height, increase in the content of chlorophyll a and b and carotenoids, free proline, the total protein, total sugars, phenols, and POD and PPO activities compared to control. Accordingly, PGPF are promising agents for applications in food processing and packaging, agricultural application, and as effective biological control against *F. oxysporum* that cause tomato wilt disease.

## Data Availability

The datasets generated during and/or analyzed during the current study are available from the corresponding author on reasonable request.

## References

[1] Wang L (2010). Effects of exogenous nitric oxide on growth and transcriptional expression of antioxidant enzyme mRNA in tomato seedlings under copper stress. Acta Horticulturae Sinica.

[2] O’Connell S (2012). High tunnel and field production of organic heirloom tomatoes: Yield, fruit quality, disease, and microclimate. HortScience.

[3] Ramakrishna W, Yadav R, Li K (2019). Plant growth promoting bacteria in agriculture: Two sides of a coin. Applied Soil Ecology.

[4] Faheed FA, Abd-Elaah GA, Mazen A (2005). Alleviation of disease effect on tomato plants by heat shock and salicylic acid infected with Alternaria solani. International Journal of Agriculture and Biology.

[5] Selim ME, El-Gammal NA (2015). Role of fusaric acid mycotoxin in pathogensis process of tomato wilt disease caused by Fusarium oxysporum. Journal of Bioprocessing & Biotechniques.

[6] Mandal S, Mallick N, Mitra A (2009). Salicylic acid-induced resistance to Fusarium oxysporum f. sp. lycopersici in tomato. Plant physiology and Biochemistry.

[7] Mohammed BL, Toama FN (2019). Biological control of Fusarium wilt in tomato by endophytic rhizobactria. Energy Procedia.

[8] Farrag A (2017). Potential impacts of elicitors to improve tomato plant disease resistance. Al Azhar Bulletin Science.

[9] Attia, M.S., et al., (2019). Eco-friendly inducers and its potential impacts to improve pear seedlings bacterial disease resistance. *Journal Biotechnology, 59*.

[10] Choudhary DK, Johri BN (2009). Interactions of Bacillus spp. and plants–with special reference to induced systemic resistance (ISR). Microbiological Research.

[11] Beneduzi A, Ambrosini A, Passaglia LMP (2012). Plant growth-promoting rhizobacteria (PGPR): Their potential as antagonists and biocontrol agents. Genetics and Molecular Biology.

[12] Ahmed, A.F., et al., *Saudi Journal of Pathology and Microbiology (SJPM) ISSN 2518–3362 (Print).*

[13] Attia MS (2020). The effective antagonistic potential of plant growth-promoting rhizobacteria against Alternaria solani-causing early blight disease in tomato plant. Scientia Horticulturae.

[14] Ali S (2020). Functional characterization of potential PGPR exhibiting broad-spectrum antifungal activity. Microbiological Research.

[15] Gorai PS (2021). Bacillus siamensis CNE6-a multifaceted plant growth promoting endophyte of Cicer arietinum L. having broad spectrum antifungal activities and host colonizing potential. Microbiological Research.

[16] Ramette A (2003). Phylogeny of HCN synthase-encoding hcnBC genes in biocontrol fluorescent pseudomonads and its relationship with host plant species and HCN synthesis ability. Molecular Plant-Microbe Interactions.

[17] Walpola BC, Yoon M-H (2012). Prospectus of phosphate solubilizing microorganisms and phosphorus availability in agricultural soils: A review. African Journal of Microbiology Research.

[18] Igiehon NO, Babalola OO (2017). Biofertilizers and sustainable agriculture: Exploring arbuscular mycorrhizal fungi. Applied Microbiology and Biotechnology.

[19] Hibar K (2007). Genetic diversity of Fusarium oxysporum populations isolated from tomato plants in Tunisia. Journal of Phytopathology.

[20] Aldinary, A.M., et al., (2021). Biocontrol of tomato Fusarium wilt disease by a new Moringa endophytic Aspergillus isolates. *Materials Today: Proceedings*

[21] Naziya B, Murali M, Amruthesh KN (2020). Plant growth-promoting fungi (PGPF) instigate plant growth and induce disease resistance in Capsicum annuum L. upon infection with Colletotrichum capsici (Syd.) Butler & Bisby. Biomolecules.

[22] Hashem AH (2020). Sustainable lipid production from oleaginous fungus Syncephalastrum racemosum using synthetic and watermelon peel waste media. Bioresource Technology Reports.

[23] Aneja, K., (2007). Experiments in microbiology, plant pathology and biotechnology. *New Age International*.

[24] Suleiman W (2018). Recruitment of Cunninghamella echinulata as an Egyptian isolate to produce unsaturated fatty acids. Research Journal Of Pharmaceutical Biological And Chemical Sciences.

[25] Hasanin MS, Hashem AH (2020). Eco-friendly, economic fungal universal medium from watermelon peel waste. Journal of Microbiological Methods.

[26] Moubasher A, Moustafa A (1970). A survey of Egyptian soil fungi with special reference to Aspergillus, Pénicillium and Penicillium-related genera. Transactions of the British Mycological Society.

[27] Khalil A (2021). Fungal endophytes from leaves of Avicennia marina growing in semi-arid environment as a promising source for bioactive compounds. Letters in Applied Microbiology.

[28] Khalil AMA, Hashem AH (2018). Morphological changes of conidiogenesis in two aspergillus species. J Pure Appl Microbiol.

[29] Hashem, A.H., et al., (2022). Isolation, identification, and statistical optimization of a psychrotolerant Mucor racemosus for sustainable lipid production. *Biomass Conversion and Biorefinery*,

[30] Hashem AH (2020). Eco-Green Conversion of Watermelon Peels to Single Cell Oils Using a Unique Oleaginous Fungus: Lichtheimia corymbifera AH13. Waste and Biomass Valorization.

[31] Hasanin, M., et al., (2021). Ecofriendly Synthesis of Biosynthesized Copper Nanoparticles with Starch-Based Nanocomposite: Antimicrobial, Antioxidant, and Anticancer Activities. *Biological Trace Element Research*, 10.1007/s12011-021-02812-0 34283366

[32] Khalil AMA, Hashem AH, Abdelaziz AM (2019). Occurrence of toxigenic Penicillium polonicum in retail green table olives from the Saudi Arabia market. Biocatalysis and Agricultural Biotechnology.

[33] Suleiman W (2018). Isolation and screening of promising oleaginous Rhizopus sp and designing of Taguchi method for increasing lipid production. Journal of Innovation in Pharmaceutical and Biological Sciences.

[34] Hashem AH (2021). Consolidated Bioprocessing of Sugarcane Bagasse to Microbial Oil by Newly Isolated Oleaginous Fungus: Mortierella wolfii. Arabian Journal for Science and Engineering.

[35] Sharaf MH (2022). Antimicrobial, Antioxidant, Cytotoxic Activities and Phytochemical Analysis of Fungal Endophytes Isolated from Ocimum Basilicum. Applied Biochemistry and Biotechnology.

[36] Hasanin MS (2020). Green ecofriendly bio-deinking of mixed office waste paper using various enzymes from Rhizopus microsporus AH3: Efficiency and characteristics. Cellulose.

[37] El-Naggar, M.E., Hasanin M., and Hashem A.H., (2021). Eco-Friendly Synthesis of Superhydrophobic Antimicrobial Film Based on Cellulose Acetate/Polycaprolactone Loaded with the Green Biosynthesized Copper Nanoparticles for Food Packaging Application. *Journal of Polymers and the Environment*,

[38] Vernon, L.P. and Seely G.R., (2014). The chlorophylls. Academic Press.

[39] Lowry OH (1951). Protein measurement with the Folin phenol reagent. Journal of biological chemistry.

[40] Irigoyen J, Einerich D, Sánchez-Díaz M (1992). Water stress induced changes in concentrations of proline and total soluble sugars in nodulated alfalfa (Medicago sativd) plants. Physiologia plantarum.

[41] Diaz, D.H. and Martin G.C., (1972).Peach seed dormancy in relation to endogenous inhibitors and applied growth substances. *American Society for Horticultural Science Journal,*

[42] Bates LS, Waldren RP, Teare I (1973). Rapid determination of free proline for water-stress studies. Plant and Soil.

[43] Srivastava, S., (1987). Peroxidase and Poly‐Phenol Oxidase in Brassica juncea Plants Infected with Macrophomina phaseolina (Tassai) Goid. and their Implication in Disease Resistance. *Journal of Phytopathology*, **120**(3): 249–254.

[44] Matta A, Dimond A (1963). Symptoms of Fusarium wilt in relation to quantity of fungus and enzyme activity in tomato stems. Phytopathology.

[45] Brownlee, K., (1952). Probit Analysis: A Statistical Treatment of the Sigmoid Response Curve. JSTOR.

[46] Snedecor, G.W. and Cochran W.G., (1982). Statistical methods. 2nd printing. Iowa State University press, Ame., USA, *507*.

[47] Vallad GE, Goodman RM (2004). Systemic acquired resistance and induced systemic resistance in conventional agriculture. Crop Science.

[48] Walters D (2005). Induced resistance: Helping plants to help themselves. Biologist.

[49] Hossain, M., F. Sultana, and S. (2017). Islam, Plant growth-promoting fungi (PGPF): phytostimulation and induced systemic resistance. *Plant-Microbe Interactions in Agro-Ecological Perspectives*, 2017: p. 135–191.

[50] Hung R, Lee S, Gupta VK (2016). Rutgers, Chapter 17 - Applications of Aspergillus in Plant Growth Promotion. New and Future Developments in Microbial Biotechnology and Bioengineering.

[51] Pandya ND (2018). Plant growth promoting potential of Aspergillus sp. NPF7, isolated from wheat rhizosphere in South Gujarat, India. Environmental Sustainability.

[52] Yoo S-J (2018). Aspergillus terreus JF27 Promotes the Growth of Tomato Plants and Induces Resistance against Pseudomonas syringae pv. tomato. Mycobiology.

[53] Imran M (2021). Multifarious functional traits of free-living rhizospheric fungi, with special reference to Aspergillus spp. isolated from North Indian soil, and their inoculation effect on plant growth. Annals of Microbiology.

[54] Badawy AA (2021). Enhancement of Seawater Stress Tolerance in Barley by the Endophytic Fungus Aspergillus ochraceus. Metabolites.

[55] Evstatieva Y (2020). Production of plant growth regulatory metabolites of Rhizopus arrhizus KB-2. Bulgarian Journal of Agricultural Science.

[56] Jovičić-Petrović J (2016). Aspergillus piperis A/5 from plum-distilling waste compost produces a complex of antifungal metabolites active against the phytopathogen Pythium aphanidermatum. Archives of Biological Sciences.

[57] Kriaa M (2015). Biocontrol of tomato plant diseases caused by Fusarium solani using a new isolated Aspergillus tubingensis CTM 507 glucose oxidase. Comptes Rendus Biologies.

[58] Peeran F, Prasad L, Kamil D (2018). Characterization of secondary metabolites from Rhizopus oryzae and its effect on plant pathogens. International Journal of Current Microbiology and Applied Sciences.

[59] Kang SW, Hong SI, Kim SW (2005). Identification of Aspergillus strain with antifungal activity against Phytophthora species. Journal of Microbiology and Biotechnology.

[60] Espinoza C (2008). Antifungal activity of several fungi against plant pathogens. Micologia Aplicada International.

[61] Frisvad JC, Larsen TO (2015). Chemodiversity in the genus Aspergillus. Applied Microbiology and Biotechnology.

[62] Meussen BJ (2012). Metabolic engineering of Rhizopus oryzae for the production of platform chemicals. Applied Microbiology and Biotechnology.

[63] Yan P-S (2004). Cyclo (L-leucyl-L-prolyl) produced by Achromobacter xylosoxidans inhibits aflatoxin production by Aspergillus parasiticus. Applied and Environment Microbiology.

[64] Orcutt, D.M., (2000). The physiology of plants under stress: soil and biotic factors. 2. John Wiley & Sons.

[65] Attia MS, Sharaf AE-MM, Zayed AS (2017). Protective Action of Some Bio-Pesticides against Early Blight Disease Caused By Alternaria Solani In Tomato Plant. JISET International Journal of Innovative Science, Engineering and Tech.

[66] Attia, M.S., et al., Comprehensive Management for Wilt Disease Caused By Fusarium Oxysporum In Tomato Plant.

[67] Attia, M.S., et al., (2016). Comprehensive Management for Wilt Disease Caused By Fusarium Oxysporum In Tomato Plant.

[68] Abdelaziz AM (2021). Protective role of zinc oxide nanoparticles based hydrogel against wilt disease of pepper plant. Biocatalysis and Agricultural Biotechnology.

[69] Alwathnani HA (2012). Evaluation of biological control potential of locally isolated antagonist fungi against Fusarium oxysporum under in vitro and pot conditions. African Journal of Microbiology Research.

[70] Ismail, A.H., et al., (2020). Thermal stress alleviating potential of endophytic fungus rhizopus oryzae inoculated to sunflower (Helianthus annuus L.) and soybean (Glycine max L.). *Pakistan Journal of Botany, 52*(5):1857–1865.

[71] Bassanezi R (2001). Accounting for photosynthetic efficiency of bean leaves with rust, angular leaf spot and anthracnose to assess crop damage. Plant Pathology.

[72] Arfan M, Athar HR, Ashraf M (2007). Does exogenous application of salicylic acid through the rooting medium modulate growth and photosynthetic capacity in two differently adapted spring wheat cultivars under salt stress?. Journal of Plant Physiology.

[73] Kyseláková H (2011). Photosynthetic alterations of pea leaves infected systemically by pea enation mosaic virus: A coordinated decrease in efficiencies of CO2 assimilation and photosystem II photochemistry. Plant Physiology and Biochemistry.

[74] Abd El‐Baky HH, El‐Baz FK, El Baroty GS (2010). Enhancing antioxidant availability in wheat grains from plants grown under seawater stress in response to microalgae extract treatments. Journal of the Science of Food and Agriculture.

[75] Nafie E (2003). The possible induction of resistance in Lupinus termis L. against Fusarium oxysporum by Streptomyces chibaensis and its mode of action: 1. Changes in certain morphological criteria and biochemical composition related to induced resistance. International Journal of Agriculture and Biology.

[76] Al-Ani RA, Adhab MA (2013). Bean Yellow Mosaic Virus (BYMV) on Broadbean: Characterization and Resistance Induced by Rhizobium leguminosarum. Journal of Pure and Applied Microbiology.

[77] Couée I (2006). Involvement of soluble sugars in reactive oxygen species balance and responses to oxidative stress in plants. Journal of Experimental Botany.

[78] Martin-Tanguy J (2001). Metabolism and function of polyamines in plants: Recent development (new approaches). Plant Growth Regulation.

[79] Lewis NG, Yamamoto E (1990). Lignin: Occurrence, biogenesis and biodegradation. Annual Review of Plant Biology.

[80] Gupta G (2001). Downy mildew induced alterations in amino acids, proline and phenols in pearl millet. Indian Journal of Plant Pathology.

[81] Al-Wakeel SA (2013). Induced systemic resistance: an innovative control method to manage branched broomrape (Orobanche ramosa L.) in tomato. European Journal of Biology.

[82] Harish S (2009). Induction of defense-related proteins by mixtures of plant growth promoting endophytic bacteria against Banana bunchy top virus. Biological Control.

